# Efficacy of Oral Remdesivir Compared to GS-441524 for Treatment of Cats with Naturally Occurring Effusive Feline Infectious Peritonitis: A Blinded, Non-Inferiority Study

**DOI:** 10.3390/v15081680

**Published:** 2023-08-01

**Authors:** Emma Cosaro, Jully Pires, Diego Castillo, Brian G. Murphy, Krystle L. Reagan

**Affiliations:** 1 William R. Pritchard Veterinary Medical Teaching Hospital, School of Veterinary Medicine, University of California, Davis, CA 95616, USA; 2 Veterinary Center for Clinical Trials, School of Veterinary Medicine, University of California, Davis, CA 95616, USA; jypires@ucdavis.edu; 3 Department of Pathology, Microbiology, and Immunology, School of Veterinary Medicine, University of California, Davis, CA 95616, USA; ldcastillo@ucdavis.edu (D.C.); bmurphy@ucdavis.edu (B.G.M.); 4 Department of Veterinary Medicine and Epidemiology, School of Veterinary Medicine, University of California, Davis, CA 95616, USA; kreagan@ucdavis.edu

**Keywords:** FIPV, coronavirus, antiviral, feline coronavirus, therapy, nucleoside analog

## Abstract

Nucleoside analogs GS-441524 and remdesivir (GS-5734) are effective in treating cats with feline infectious peritonitis (FIP). However, no studies have compared the efficacy between antiviral medications. The objective of this study was to evaluate the efficacy of orally administered GS-442514 (12.5–15 mg/kg) compared to orally administered remdesivir (25–30 mg/kg) in a double-blinded non-inferiority trial. Eighteen cats with effusive FIP were prospectively enrolled and randomly assigned to receive either GS-442514 or remdesivir. Cats were treated daily for 12 weeks and evaluated at week 0, 12, and 16. Survival and disease remission at week 16 were compared between groups. Five of 9 (55%) cats treated GS-441524 and 7/9 (77%) cats treated with remdesivir survived, with no difference in survival rate (*p* = 0.2). Remdesivir fulfilled the criteria for non-inferiority with a difference in survival of 22% (90% CI; −13.5–57.5%). Three of the 18 cats died within 48 h of enrollment. Excluding these cats, 5/6 (83%) of the cats treated with GS-441524 and 7/9 (77%) of the cats treated with remdesivir survived. These findings suggest that both orally administered GS-441524 and remdesivir are safe and effective anti-viral medications for the treatment of effusive FIP. Further optimization of the first 48 h of treatment is needed.

## 1. Introduction

Feline coronavirus (FCoV) is a large single-stranded RNA virus in the family coronaviridae and is found in cats worldwide [1]. Feline infectious peritonitis (FIP) is a disease that results from a mutation or set of mutations in the ubiquitous, non-pathogenic biotype of FCoV referred to as feline enteric coronavirus (FECV). Mutations confer a cellular tropism switch from enterocytes to monocytes and macrophage, facilitating systemic distribution of the coronavirus. The prevalence of FCoV is high, with approximately 20% of cats living in private households and 87% of purebred cats housed in catteries testing seropositive for infection with FCoV [2]. Cats infected with FCoV often have no clinical signs or will have mild self-limiting gastroenteritis, with diarrhea being the most common clinical sign reported. Mutations in the gene encoding the viral surface receptor, Spike (S), are associated with an expansion of tropism from enterocytes to macrophages, resulting in systemic viral dissemination and immune-mediated perivasculitis and pyogranulomatous inflammation [2,3]. Approximately 5–12% of cats infected with FCoV will develop these mutations in the S gene and develop FIP [3]. FIP presents as a spectrum disease with two classic forms, the effusive or “wet” form which presents with cavitary effusions or the “dry” or non-effusive form which presents as pyogranulomatous infiltrates affecting the liver, kidneys, lymph nodes, central nervous system, or ocular structures. Without treatment, FIP is essentially 100% fatal [1,4].

Safe and effective antiviral therapies for cats with FIP have been identified, and the nucleoside analog GS-441524 has been used to treat thousands of cats worldwide with FIP [5,6,7,8,9,10,11,12]. In virus-infected cells, GS-441524 is incorporated into the nascent viral RNA strand resulting in the premature termination of viral RNA synthesis and halting viral replication. GS-441524 is the metabolite of the antiviral drug remdesivir (GS-5734) used to treat people with SARS-CoV-2 infections [13,14,15], and both drugs yield the same active metabolite in the host cell [14]. Remdesivir has been demonstrated to have equivalent efficacy to GS-441524 in suppressing FIPV replication in in vitro studies and has been used in combination with GS-441524 to treat cats with FIP [11,12,16]. However, no prospective studies have evaluated its efficacy and outcomes for cats treated with remdesivir alone compared to GS-441524 alone. The objective of this study was to compare the efficacy of oral remdesivir used to treat naturally occurring, effusive FIP in a prospective, blinded, non-inferiority clinical trial to cats treated with oral GS-441524.

## 2. Materials and Methods

### 2.1. Study Design

A single-center, prospective, double-blinded, longitudinal trial with two parallel treatment groups was performed. Cats diagnosed with naturally occurring, effusive FIP and met the study enrollment criteria were randomly assigned to one of two antiviral treatment groups, GS-441524 or remdesivir, as outlined below. The study was performed with caretaker-provided informed consent prior and with the approval of the University of California Davis Institutional Animal Care and Use Committee (IACUC 22773, approval date 2 May 2022).

### 2.2. Inclusion Criteria

A diagnosis of effusive FIP was made if pleural, peritoneal, or pericardial effusion was noted and (1) there was detection of FCoV antigen by immunohistochemistry in the context of biopsied granulomatous lesions as determined by a veterinary pathologist [17,18] or (2) detection of FCoV antigen by immunocytochemistry within nucleated cells upon cytologic evaluation of effusions as determined by a veterinary pathologist [19,20,21]. Alternatively, cats were diagnosed with effusive FIP if (3) FCoV RNA was detected within the effusion using real time RT-PCR performed by a commercial laboratory or UC Davis laboratory [22,23,24,25,26] and demonstrated ≥3 of the following examination or clinicopathologic features; fever documented on two occasions over 12 h apart (rectal temperature > 102.5 °F), lymphocytes below the lower limit of normal, globulins above the upper limit of normal, albumin:globulin ratio < 0.6, bilirubin above the upper limit of normal, effusion with total protein > 3.5 g/dL with cytology assessed by a veterinary clinical pathologist to be consistent with FIP, or positive FCoV antibody serology tested through a commercial laboratory or the UC Davis Clinical Diagnostic Laboratory [27,28,29,30,31].

To be included in the study, cats were required to have a negative feline leukemia virus antigen and feline immunodeficiency virus antibody test within the preceding one month. Cats were excluded from the clinical trial if they had been treated with any anti-coronaviral medications before enrollment, if the cat or caretaker was unable to cooperate fully with the requirements of the study protocol, including the schedule of assessments, or was likely to be non-compliant with any study requirements. Further, cats were excluded if they had a neutrophil count < 2000/μL, platelet count < 75,000/μL, creatinine above the upper limit of normal, ALT > twice the upper limit of normal, if they were clinically assessed as needing a blood transfusion, if they were hypothermic (rectal temperature < 97 °F), if they were not able to take *per os* medications, or if the study veterinarian did not have a reasonable expectation that the patient would live for 48 h.

### 2.3. Treatment Groups and Drug Dosing Protocol

Cats were randomly assigned (1:1) to receive either oral GS-441524 or oral remdesivir using the envelope method [32]. Study veterinarians and cat caretakers were blinded to the treatment group. Antiviral compounds were sourced from Natural Micron Pharm Tech (NM PharmTech; Tai’an, China) and were compounded into identical gelatin capsules. *In vitro* assays determined that both GS-441524 and remdesivir were effective in inhibiting FIPV replication with EC50 values comparable to previously published values [16]. Bioequivalent dosing based on the compound molecular weight was utilized, with a dosing range of 12.5–15 mg/kg PO once daily for GS-441524 and 25–30 mg/kg PO once daily for remdesivir for 12 weeks. Cats were weighed weekly during the study and dosing was adjusted to remain within the study dosing range for the duration of the study.

### 2.4. Study Protocol

**Figure 1:**
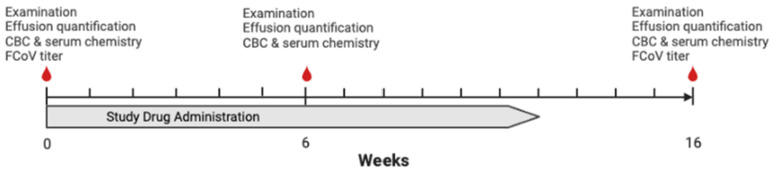
Study timeline indicating timing of evaluation and study drug administration. Weight was recorded weekly for the study duration. (Figure made with Biorender).

Cats were evaluated during three visits, week 0, week 6, and week 16 (Figure 1). At each visit, a physical examination was performed by a study veterinarian (EC, KLR). At enrollment, a complete blood count (Advia 120; Siemens, Munich, Germany), serum chemistry panel (Cobas c501/6000; Roche, Basel, Switzerland), FCoV antibody titers (Clinical Virology and Immunology Laboratory, UC Davis Veterinary Medical Teaching Hospital), and effusion analysis and cytology (Clinical Diagnostic Laboratory, UC Davis Veterinary Medical Teaching Hospital) were performed if diagnostics had not been performed within one week before enrollment through a reference laboratory. Baseline FCoV antibody titers were recorded as an endpoint titer if available, the highest positive titer tested was recorded, or as positive or negative if no titer was reported. Effusion was quantified at each visit. Pleural and pericardial effusion volumes were recorded using a thoracic FAST scan. Unilateral or bilateral fluid accumulation was noted and quantified by recording the cm of fluid noted in the area of greatest accumulation [33]. Abdominal effusion was recorded using an abdominal FAST scan (AFS). An AFS score of 0 to 4 was assigned as previously described [34]. A CBC and serum chemistry panel were performed at week 6 and week 16, and FCoV antibody titer were performed at week 16 (Clinical Diagnostic Laboratory, UC Davis Veterinary Medical Teaching Hospital). Caretaker reported weight was recorded weekly. Additional visits and supportive care could be prescribed at the discretion of the attending veterinarian. The caretaker was instructed that they could withdraw their cat from the study at any time. All drug-associated adverse events were recorded. Cats that died or were humanely euthanized during the study were assessed with a postmortem examination when possible.

### 2.5. Survival Evaluation and Statistical Analysis

A non-inferiority margin of 15% was selected based on perceived clinically relevant changes in outcomes. The study was designed to have more than 90% power and alpha of 5% to detect non-inferiority of remdesivir compared to GS-441524 using previously described methods [35]. A minimum of eight individuals were required to test this hypothesis. Up to two additional cats were enrolled in each group to account for a potential caretaker withdrawal rate of up to 20%. A primary outcome measure of survival and disease remission as defined by absence of effusion and resolution of all clinical signs at 16 weeks was established. Overall survival for all cats in each group with 95% confidence intervals for each group were calculated with all cats in the per protocol treatment groups. Additionally, survival rates were also calculated with cats that died <2 days post enrollment being censored from analysis [12]. Survival curves were generated, and the groups were compared with a logrank (Mantel–Cox) test. Descriptive statistics were utilized to summarize clinicopathologic parameters. Normality was assessed using a Shapiro–Wilk normality test and parametric or non-parametric testing was utilized to compare parameters at baseline. Clinicopathologic parameters were evaluated using linear mixed-effects models due to the repeated measures (Prism Version 10.0.0, GraphPad).

### 2.6. Necropsy and Tissue Analyses

If allowed by the caretaker, cats that succumbed to disease during the study had a timely and complete necropsy examination performed within 24 h of death. A complete set of tissues, including the brain, were collected in 10% buffered formalin for a minimum of 24 h. Tissues were then trimmed, embedded in paraffin, and routinely processed for histological examination. Tissues with pyogranulomatous perivascular inflammation, characteristic of FIP, were further evaluated by an immunohistochemistry (IHC) assay to detect coronaviral antigen (FIPV3-70, Custom Monoclonals International, Sacramento, CA, USA) [36]. Histology and immunohistochemical stains were performed at the UC Davis Veterinary Histology Laboratory and interpreted by a single pathologist (BM). Each IHC stain was performed in parallel with known FIP-positive feline control tissue and an irrelevant isotype-control antibody as the negative control.

## 3. Results

### 3.1. Cat Demographics and Diagnosis

**Figure 2:**
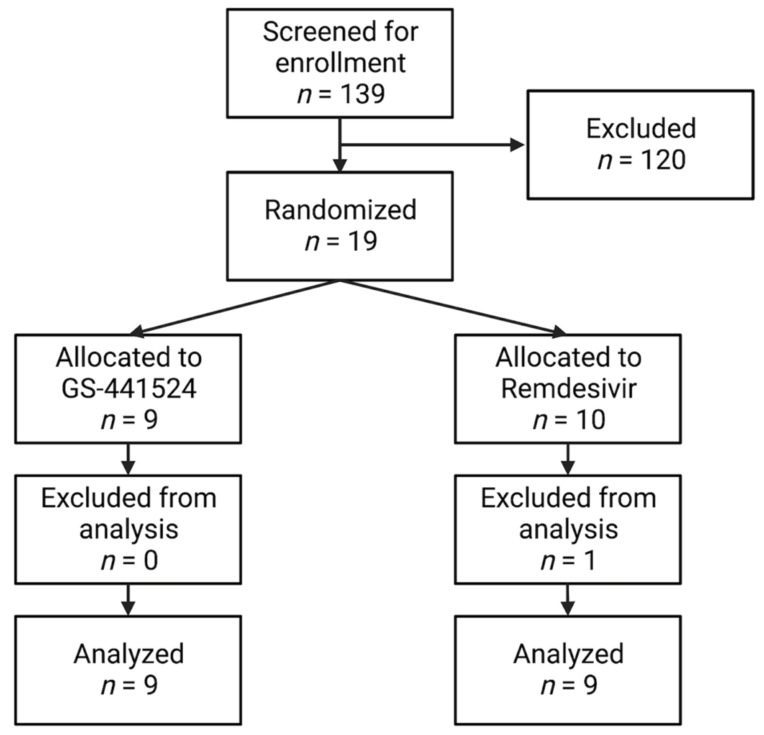
Consort diagram describing study screening, exclusions, intention to treat animals that were allocated to the antiviral treatment group, and cats that were excluded from the final analysis. (Image made with Biorender).

One-hundred and thirty-nine client-owned cats were screened for enrollment. One-hundred and twenty cats were excluded because they did not meet diagnostic criteria, they had already been administered anti-viral therapy, no effusion was noted, or the caretakers could not comply with the study criteria. One cat was initially allocated to the remdesivir group, but upon record review did not meet enrollment criteria, so was subsequently excluded from the analysis. This cat died on treatment day 2 and a necropsy was performed and described below. Eighteen cats met the study criteria and were randomly allocated to a treatment group, with nine cats in the remdesivir group and nine cats in the GS-441524 group (Figure 2). Baseline demographics data for the cats is summarized in Table 1. Purebred cats include two Bengals, two Maine Coons, and one each of Ragdoll, Siamese, and Sphinx cats. All cats had a positive real-time RT-PCR result for FCoV on effusion and fulfilled ≥3 other study criteria to meet inclusion criteria. This included 10 cats with a fever, 10 cats with hyperglobulinemia, 16 cats with an a:g ratio < 0.6, 11 cats with hyperbilirubinemia, 10 cats with lymphopenia, and 17 cats with an effusion cytology analysis. All cats were FeLV and FIV negative by point-of-care testing.

### 3.2. Clinicopathologic Findings at Diagnosis

Clinicopathologic variables at the time of study enrollment are presented in Table 2. Twelve cats had abdominal effusion, five had pleural effusion, and one cat had pericardial effusion observed with an ultrasonographic evaluation. At the time of enrollment, all cats with abdominal effusion had an AFAST scores of 4/4. The abdominal effusion had a median total nucleated cell count of 2860 cells/μL (range 200–26,960 cells/μL) and median total protein of 6.5 g/dL (range 4.5–8.4 g/dL). The pleural effusion had a median total nucleated cell count of 1929 cells/μL (range 1280–11,660 cells/μL) and median total protein of 6.8 g/dL (range 5.0–7.6 g/dL). One cat had pericardial effusion, and the total nucleated cell count was 10,440 cells/μL with a total protein of 5.7 g/dL. Fluid cytology analyses did not identify any other etiologic diagnosis in any cat enrolled in the study.

### 3.3. Concurrent Therapy, Co-Morbidities, and Adverse Effects

In GS-441524 group, no cats experienced adverse events that necessitated stopping therapy. One cat had diarrhea prior to enrollment in the study that persisted during the study and was treated with a probiotic supplement and calcium aluminosilicate clay until resolution of signs. Two additional cats developed self-limiting diarrhea and vomiting during the study. One cat developed ocular discharge and was treated with ofloxacin 0.3% ophthalmic drops and lysine supplement orally. Another cat in this group developed signs of an upper respiratory infection including sneezing and nasal discharge and was treated with oral azithromycin. One cat had chronic otitis externa before study enrollment and was treated with topical thiabendazole, dexamethasone, neomycin solution. This cat also had chronic gingivostomatitis that was not treated during the study period. One cat was treated with transdermal mirtazapine. One cat was treated with intravenous lactated ringers, dextrose supplementation, pantoprazole, and ampicillin/sulbactam.

For cats treated with GS-441524, no clinically relevant biochemical or hematologic abnormalities were noted that necessitated stopping therapy. Three cats in this group developed hypocholesterolemia (77 mg/dL, 85 mg/dL, 68 mg/dL; reference range 89–248 mg/dL) at week 6 which resolved in all but one cat (54 mg/dL at week 16). Hyperphosphatemia and elevated ALP above baseline values were noted in four juvenile cats. One cat developed a lymphopenia (481/uL; reference range 1000–7000/uL), basophilia (241/uL; reference range 0–200/uL), and monocytosis (722/uL; reference range 500–600/uL) of which only basophilia was persistent at week 16 (171/uL). One cat developed eosinophilia (2112/uL; reference range 150–1100/uL) which was persistent but improved at week 16 (1840/uL). One cat developed a mild anemia at week 6 (HCT 29.0%; reference range 30–50%) which resolved at week 16 (34.4%). One cat developed elevated BUN at week 16 (BUN 37 mg/dL; reference range 18–33 mg/dL). No hepatocellular enzyme elevations were noted during the study period.

In the remdesivir group, no cats experienced adverse events that necessitated stopping therapy. Three cats developed self-limiting diarrhea. Two cats developed clinical signs of an upper respiratory tract infection, one of which was treated with ofloxacin 0.3% ophthalmic drops. One cat was diagnosed with restrictive pericarditis and underwent pericardiectomy on study day 20. That cat received anesthetic medications, ampicillin/sulbactam, robenacoxib, and fentanyl in the peri-operative period. One cat in this group was treated with methylprednisolone acetate three weeks before enrollment. General supportive therapies for cats in this group included subcutaneous lactated ringers in one cat, and transdermal mirtazapine in three cats. One cat was treated with oral fenbendazole. One cat developed hyperesthesia and was treated with gabapentin.

No clinically relevant biochemical or hematologic abnormalities were noted in cats treated with remdesivir. Hyperphosphatemia and elevated ALP above baseline values were noted in five juvenile cats. Two cats had new elevations in creatine kinase at week 16 (325 IU/L and 380 IU/L; reference range 73–260 IU/L). One cat developed hypocholesterolemia at week 6 (98 mg/dL; reference range 89–248 mg/dL) which resolved at week 16. One cat developed a thrombocytosis at week 6 (517,000 platelets; reference range 180,000–500,000). One cat had elevated BUN at week 16 (34 mg/dL; reference range 18–33). No hepatocellular enzyme elevations were noted during the study period.

### 3.4. Survival Analysis

In the GS-441524 treatment group, the average dose throughout the study was 13.6 mg/kg (range 12.6–15 mg/kg). Four of the nine cats died during the study period. Three cats died or were euthanized in the first 48 h of starting therapy, and one died on day 4. Necropsy findings were available for one cat that died 24 h after enrollment and are described below. Overall survival in this group was 55.6%, and for cats that survived at least 48 h of treatment, the survival was 5/6 (83%). All surviving cats were determined to be in clinical remission at 16 weeks based on the absence of effusion and the resolution of clinical signs. Two cats in this group had an albumin:globulin ratio > 0.6 at 16 weeks but continue to be clinically well at four months and two months post-study conclusion. A median survival time was not reached. All the surviving cats continued to be clinically well at the time of journal submission with a time post last antiviral treatment dose of 136–283 days. No cats in this group were treated for relapse of their disease.

In the remdesivir treatment group, the average dose throughout the study was 25.9 mg/kg (range 23.2–27.9 mg/kg). Two of the 9 cats died during the study period. One cat was euthanized on treatment day 24 due to complications of restrictive pericarditis and post-operative pericardial stripping procedures and one cat died on treatment day 4 at home. A necropsy evaluation was performed on the cat that died on treatment day 4. All other cats survived and were in remission based on the absence of effusion and the resolution of clinical signs resulting in an overall survival of 7/9 (77.8%) in this group. A median survival time was not reached. One cat in this treatment group developed seizures at 139 days post-conclusion of treatment. This cat was euthanized, and no necropsy examination was performed. All the remaining surviving cats continued to be clinically well at the time of journal submission with a time post last antiviral treatment dose of 97–265 days. No cats in this group were treated for relapse of their disease.

**Figure 3:**
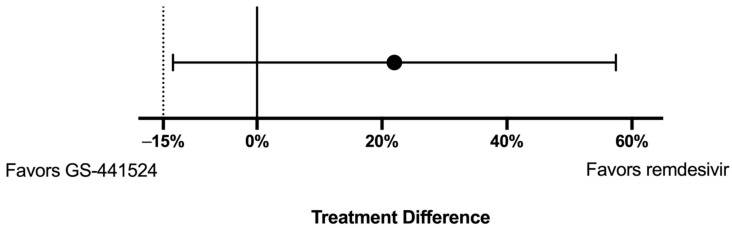
Treatment difference. The difference in survival rate of remdesivir minus GS-441524 (black dot) displayed with the 95% confidence interval of the difference in survival. A solid vertical line is placed at 0% difference in survival and a dotted vertical line represents the 15% non-inferiority limit.

**Figure 4:**
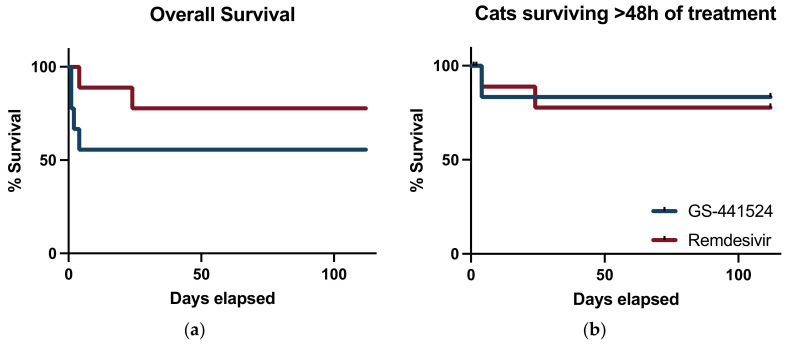
Survival curves: (**a**) survival curve that displays all-cause mortality; and (**b**) survival curve with cats that succumbed to disease within the first 48 h of therapy censored from analysis. Cats treated with GS-441524 are represented by the blue line and cats treated with remdesivir are represented by the red line.

The difference in proportion of cats that did not survive between the two treatment groups was 22% (90% CI; −13.5–57.5%) and treatment with remdesivir met the *a priori* set non-inferiority limit of −15%. (Figure 3) There was no difference noted between the survival curves for each treatment group when assessing all-cause mortality (*p* = 0.24) (Figure 4a). When cats that died within 48 h of enrollment were considered censored subjects, there were no difference between survival between groups (*p* = 0.82) (Figure 4b).

Postmortem evaluations were performed for three non-survivors in the study. A five-month-old spayed female domestic shorthair cat that died on treatment day 1 in the GS-441524 group was evaluated. Gross and microscopic lesions identified included: icterus, severe abdominal effusion, and pyogranulomatous inflammation of multiple abdominal organs (intestinal serositis, splenic capsulitis, pancreatitis, hepatitis, and mesenteric lymphadenitis). An IHC assay was negative for detectable coronavirus antigens.

**Figure 5:**
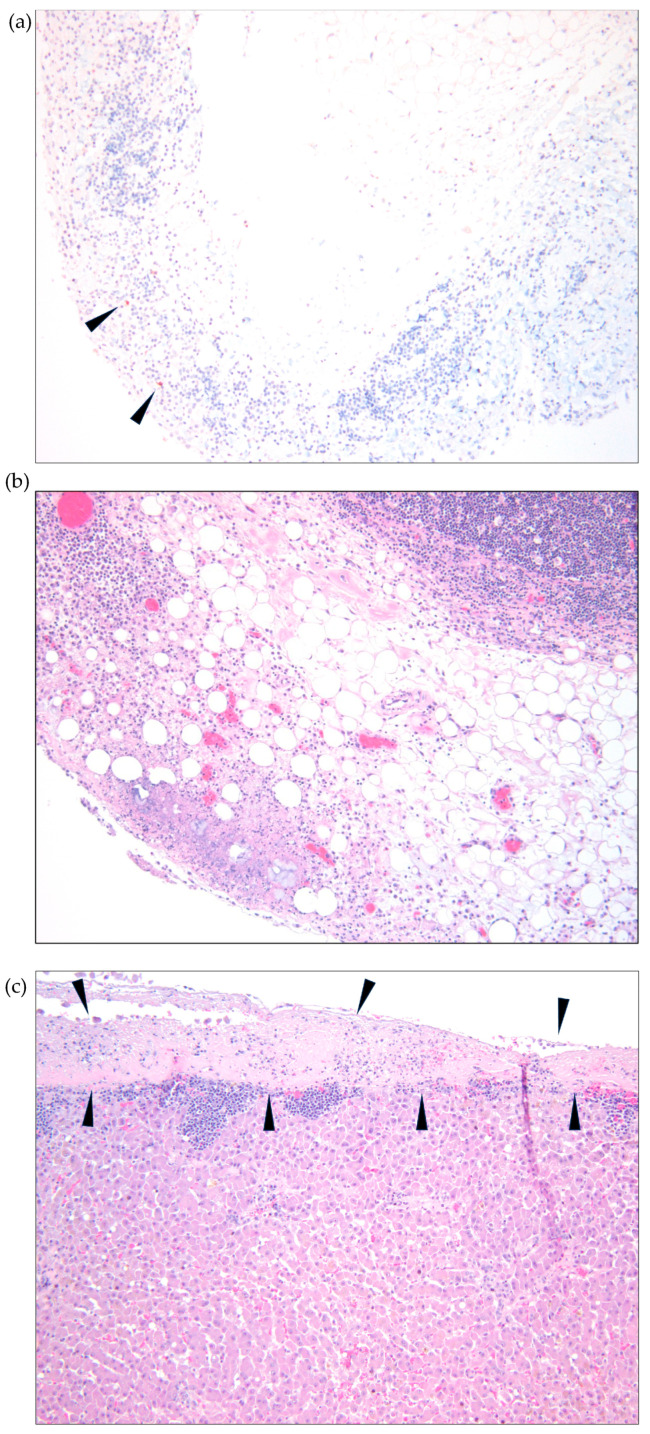
(**a**) Lymph node exhibiting minimal positive IHC coronaviral antigen; (**b**) lymph node histopathology consistent with pyogranulomatous inflammation; and (**c**) liver histopathology exhibiting inflammation of the capsule (capsulitis).

An 11-year-old spayed female domestic shorthair cat that died on treatment day 4 in the remdesivir group was also evaluated postmortem. Gross and microscopic lesions that were identified included: mild icterus, moderate abdominal effusion, fibrinous peritonitis, hepatitis, splenitis and lymphadenitis (Figure 5a). Phlebitis was identified in the vessels surrounding the abdominal lymph nodes and both the liver and the heart were smaller than normal (microhepatica and microcardia). Despite the presence of florid inflammatory lesions, only small numbers of intralesional macrophages demonstrated intracytoplasmic coronaviral antigens using IHC assays (Figure 5a,b).

A necropsy examination was also performed on a two-month-old male domestic short hair kitten that died on treatment day 2 in the remdesivir arm of the study. However, this cat was excluded from the overall survival analyses because it was determined that the cat did not meet the initial enrollment criteria. Gross and microscopic lesions included: ascites, intestinal serositis, hepatitis, pancreatitis, and ocular choroiditis. Coronaviral IHC assays revealed small amounts of intracytoplasmic coronaviral antigens in macrophages within lesions in the liver, intestinal serosa, pancreas, mesentery, and eye. In addition to the lesions consistent with FIP, this kitten also had lesions of bacterial hepatitis, embolic pneumonia, hypertrophic cardiomyopathy, and obstipation of the colon.

### 3.5. Secondary Outcomes

**Figure 6:**
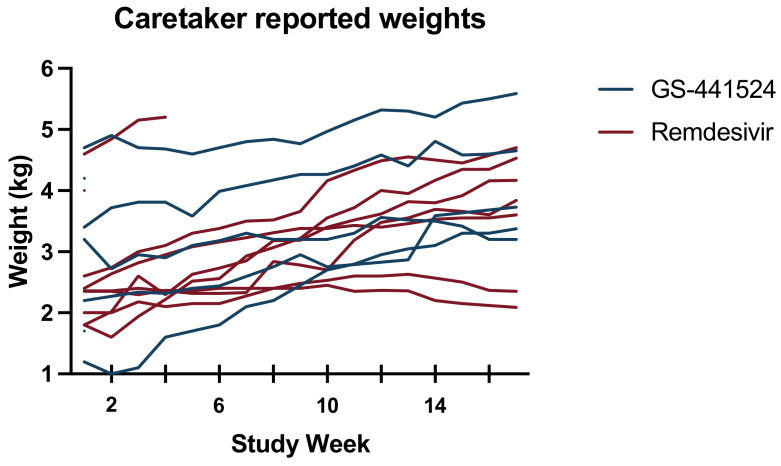
Weight (kg) measured weekly for individual study cats. Truncated lines represent non-surviving cats. Cats in the GS-441524 treatment group are represented by blue lines and cats in the remdesivir treatment group are represented by red lines.

**Figure 7:**
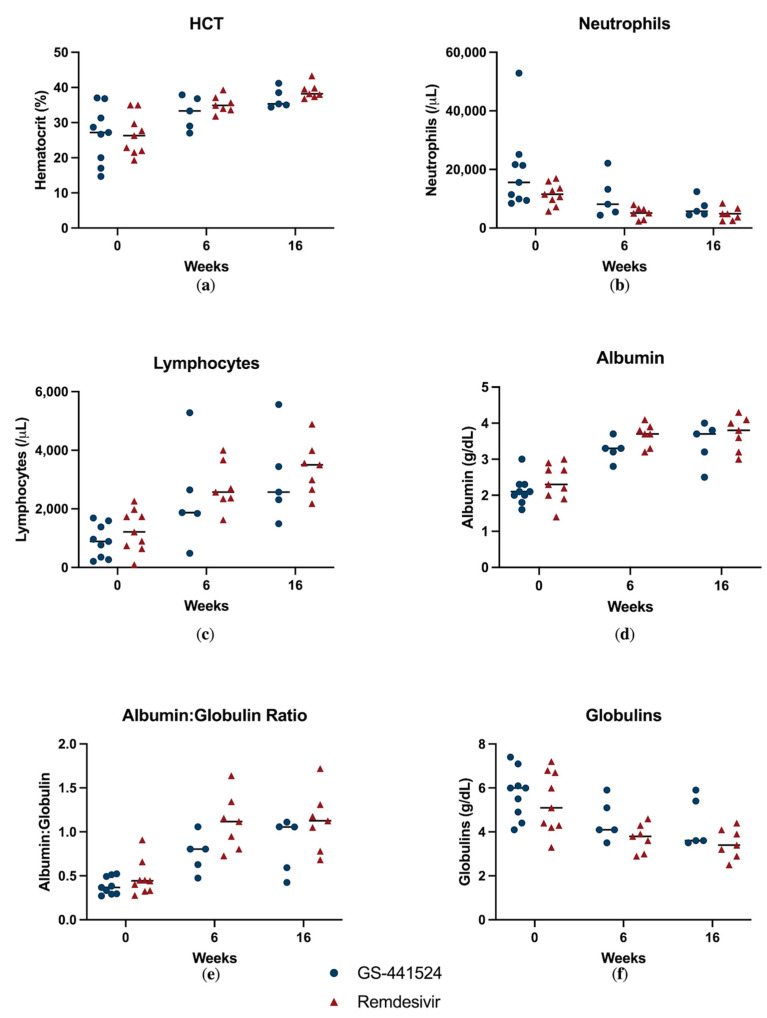
Clinicopathologic features throughout the study period including: (**a**) hematocrit; (**b**) neutrophil count; (**c**) lymphocyte count; (**d**) serum albumin concentration; (**e**) serum albumin:globulin ratio; and (**f**) serum globulin concentrations. Cats treated with GS-441524 are represented by blue circles and cats treated with remdesivir with red triangles. The median value represented with a horizonal black line.

Effusion resolved in all the surviving cats by week 6, and there was no difference between treatment groups. The caretaker reported that weekly weights changed significantly over time (*p* = 0.0002), but there was no significant factor difference between the antiviral treatment groups (Figure 6). Biochemical parameters throughout the study period are reported in Figure 7. All cats with a documented hyperglobulinemia or hyperbilirubinemia had resolution of these abnormalities by week 16, except for one cat with concurrent gingivostomatitis and upper respiratory infection that had an elevated globulins (5.9 g/dL; reference range 2.8–5.4 g/dL) in the GS-441524 group. Further, in a mixed-effects model, time was a significant factor for hematocrit (*p* = 0.0004), neutrophil count (*p* = 0.0114), lymphocyte count (*p* = 0.0002), serum albumin concentration (*p* < 0.0001), serum globulin concentration (*p* = 0.0016), the albumin:globulin ratio (*p* < 0.0001), and serum bilirubin concentration (*p* = 0.0081). A significant factor effect was noted between anti-viral treatment groups for albumin:globulin ratio (*p* = 0.03), but none of the other evaluated biochemical parameters.

**Figure 8:**
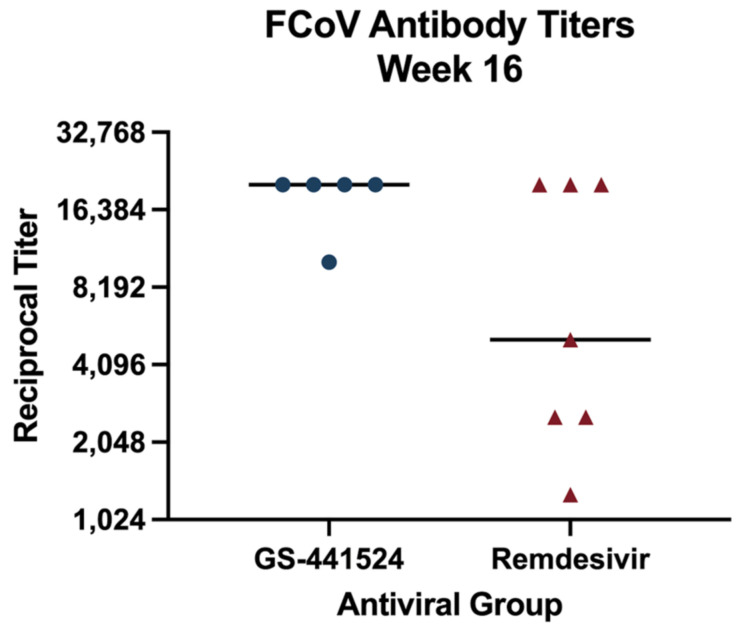
Feline coronavirus antibody serology at study week 16, presented as a reciprocal endpoint titer. Specimens were not tested at dilutions higher than 1:20,480. Cats in the GS-441524 treatment group are in represented by blue dots, and cats in the remdesivir treatment group are represented by red triangles. The black horizontal line represents the median antibody titer.

Feline coronavirus serology was performed in 14/18 cats at baseline, and all were positive. In the GS-441524 treatment group, this included five cats with titers ≥1:20,480 and one cat with a titer ≥1:5120. In the remdesivir treatment group, this included five cats with titers ≥1:20,480, two cats each with titers ≥1:12,800, and one cat with a titer ≥1:5120. At study week 16, all surviving cats had serology performed in the same laboratory, and all cats had positive titers (Figure 8). There was no difference in FCoV titers between treatment groups at week 16.

## 4. Discussion

This randomized, double-blinded treatment trial for cats with naturally occurring effusive FIP demonstrated that oral administration of remdesivir (25–30 mg/kg PO once daily) is non-inferior to treatment with oral GS-441524 (12.5–15 mg/kg PO once daily) at bioequivalent dosing. Overall survival did not differ between the treatment groups in this study and overall prognosis for remission was good for cats that survived >48 h of antiviral therapy. This study demonstrated survival rates near 80% for cats treated with either remdesivir or GS-441524, if they survived the first two days of therapy. The antiviral treatment group was not significantly associated with changes to hematocrit, albumin, or globulins during treatment, indicating that either medication can be effectively utilized to treat cats with naturally occurring effusive FIP.

In this study, overall survival was 55% for cats treated with oral GS-441524 and 77% for cats treated with oral remdesivir. There was no statistical difference noted in the survival rates between these two treatment groups, but these rates are lower than previously reported survival rates for cats undergoing antiviral treatment for FIP. In an experimental FIP feline model, 100% (10/10) of cats had complete resolution of disease when treated with GS-441524 [5]. Further, a study evaluating GS-441524 in client-owned, naturally infected cats, including those with effusive and non-effusive FIP, also showed a favorable response [6]. Thirty-one cats in that study were treated with GS-441524 at a dose of 2 mg/kg SC q24h for 12 weeks with a dose escalation to 4 mg/kg q24h in five cats. At the conclusion of the study, 93% of the cats with the effusive form of FIP were in clinical remission [5]. Subsequent to the publication of these studies, many owners have turned to a variety of unlicensed antiviral therapies purchased online and administered at home, frequently without veterinary guidance. A recent study surveying an online community of owners using GS-441524 showed that 88.2% of owners reported improvements in clinical signs within one week of starting the drug and 96.7% of cats being treated were alive at the time of the study survey [7]. Unlicensed therapies available on the Internet are thought to be similar in chemical composition to the antiviral drug GS-441524, and a clinical trial evaluating the effectiveness of these drugs in a controlled setting revealed clinical remission in 100% (18/18) of cats [9]. Larger-scale studies have now demonstrated remission rates of 80–94% [10,11,12]. The reasons for the lower survival rates in our study may reflect a population of animals that are more medically compromised at the start of anti-viral therapy as a reflection of the patient population at our tertiary referral hospital. Often our patients need to travel significant distances or present first to a local veterinarian, which may in turn result in delays in starting antiviral therapy.

Another potential contribution to the lower-than-expected survival rate in this study is the route of antiviral administration. Cats in this study received exclusively oral antiviral therapy. While some previous studies utilized oral antiviral therapy, the large-scale studies allowed for initial administration of parenteral antiviral therapy with the hypothesis that this may result in more rapid achievement of therapeutic drug levels when compared to animals receiving exclusively oral therapy. Pharmacokinetic studies have demonstrated that oral dosing of GS-441524 and remdesivir yield therapeutic plasma drug concentrations exceeding the EC50 values over 24 h [16]. Three of the non-surviving cats underwent necropsy analyses, on days one, two, and four of antiviral therapy. Two cats had evidence of intralesional FCoV antigen detected using IHC, but at very low levels, and in one cat IHC staining did not reveal lesions associated FCoV antigen. This very low level of antigen detection after short courses of antiviral therapy indicates that the medications reach effective concentrations and rapidly decrease viral burdens. However, severe inflammatory responses were still present and likely contributed to the demise of these patients. Studies assessing differences between outcomes for cats with naturally occurring FIP receiving oral and parenterally administered antiviral therapy should be pursued to establish the optimal treatment protocol.

Historically, the diagnosis of FIP relied upon the gold standard identification of FCoV antigen with IHC upon histopathology of affected tissues. However, this methodology often requires invasive procedures to procure biopsy specimens and is not attainable in moribund animals. Therefore, alternative diagnostic strategies have been established [22,23,24,26]. In this study, we relied upon identification of FCoV nucleic acid with RT-PCR from an affected lesion, such as effusion in the context of clinical diseases consistent with FIP. A limitation of this study is that the RT-PCR performed at the time of diagnosis was not standardized and performed in the same laboratory. Therefore, comparisons of viral nucleic acid load between cats cannot be performed.

Dosing strategies for GS-441524 in the literature are variable, ranging from 2–4 mg/kg SC or 5–15 mg/kg orally once daily for 12 weeks [6,7,9,10,11,12]. However, it is difficult to fully understand the dosing in some previous trials, as the compound administered is often of an unknown concentration and the true dose is difficult to elucidate. The dosing strategy utilized in this study was based on pharmacokinetic studies that demonstrated an oral remdesivir dose of 25 mg/kg once daily achieved therapeutic plasma concentrations [16]. From there, a dose of GS-441524 of 12.5 mg/kg was determined as the molecular weight of half that of remdesivir. Therefore, bioequivalent dosing was achieved, removing this factor impacting differences in patient outcomes. A dosing range was utilized for ease of drug-compounding and the dose was adjusted weekly based on patient weight to ensure the antiviral dose remained as close to the dosing target as possible. Further prospective studies are needed to fully investigate oral dosing strategies of remdesivir and GS-441524 for cats with FIP, including the necessary duration of therapy.

In surviving cats, all effusion was resolved by the week 6 visit. Time to resolution of effusion comparisons between study groups could not be completed with this study design, as ultrasonographic evaluation would need to have been completed more frequently to determine when it resolved. Clinicopathologic parameters, including hematocrit, lymphocyte count, serum proteins, and bilirubin all largely normalized during the study in both treatment groups. There was no significant treatment effect noted in this study regarding the anti-viral drug administered, except for the albumin:globulin ratio. One cat in the GS-441524 group had persistently elevated globulins, without any other remaining signs of ongoing FIP. This cat was also diagnosed with chronic upper respiratory tract infections and chronic gingivostomatitis. At the time of manuscript submission, the caretakers had not reported any relapse of FIP clinical signs; therefore, this finding of a decreased albumin:globulin ratio in the GS-441524 group likely reflects a type 1 error rather than a true drug effect. All cats remained seropositive at week 16 and there was no difference in titers between antiviral treatment groups.

One cat in the remdesivir treatment group developed seizures and was euthanized 139 days after completion of treatment. This cat had a positive response to therapy during the treatment period and the observation period post-treatment, with normalization of globulins, fever, peritoneal effusion. However, this cat developed hyperesthesia syndrome post-treatment and then developed seizures and there is concern this may be a manifestation of a neurologic form of FIP. A necropsy evaluation was not allowed, so confirmation of this suspicion was not possible. All other cats in both treatment groups have continued to remain in clinical remission of their disease. If it is assumed that this cat experienced a relapse of FIP, the relapse rate in this study (overall 8.3%) is similar to other studies that have a demonstrated relapse rate of 3.5–23% of cats treated with either GS-441524, remdesivir, or a combination of the medications [6,7,11,12].

No clinically relevant adverse events were noted in this study. In people, rare reports of liver enzyme elevations have been observed in remdesivir clinical trials [37]. However, this was not observed in our study for cats receiving the medication daily for 84 days. This is similar to other studies that have treated cats with GS-441524, remdesivir, or a combination of the medications [6,7,11,12]. Other, less-apparent toxicities, such as impairment of fetal devolvement were not investigated in this study [38].

Study limitations included a lack of continual monitoring in the first weeks of the study. Cats were only examined three times during the study protocol to limit stress to the animals and associated costs. This prevented the collection of more granular details for some secondary-outcome measures such as the resolution of effusion. Cats received antivirals dosed within a dosing window corresponding to standard compounding-sized capsules rather than custom-made capsules that provided a consistent dose throughout the study; therefore, it is possible that there were dose-related effects between the groups since they were not individually compounded for patient weight. The collection of clinicopathologic data at baseline was not standardized and there may have been interlaboratory variation for some baseline data, especially FCoV antibody titers. If a patient had laboratory work that was performed with a reference laboratory within one week of presentation for study screening, further blood collection was not performed due to the severe illness, small blood-circulating volume, and marked anemia noted in many of the patients. Complete blood count and chemistry panel performed at study weeks 6 and 16 were all performed in the same diagnostic laboratory for consistency in evaluating study outcomes. Several of the cats enrolled in the study that succumbed to disease did not have follow-up postmortem examinations performed, limiting the ability to fully assess the causes of death in those patients.

## 5. Conclusions

Orally administered remdesivir at 25–30 mg/kg by mouth once daily for 12 weeks was non-inferior to orally administered GS-441524 at a bioequivalent dose of 12.5–15 mg/kg by mouth once daily for 12 weeks in cats with naturally occurring effusive FIP. These medications were well-tolerated without any clinically applicable adverse events. Mortality rates in this study were higher than in some previous reports and causes for this difference should be further evaluated with rigorous, prospective, controlled clinical trials evaluating the efficacy of oral versus parenteral antiviral therapy.

## Figures and Tables

**Table 1:** Baseline demographics.

	All Cats	GS-441524 Allocated Cats	Remdesivir Allocated Cats
Number	18	9	9
Purebred	6	3	3
Age (months) *	6 (3–164)	7 (3–164)	6 (3–140)
Sex			
Intact Female	3	2	1
Spayed Female	8	5	3
Intact Male	4	1	3
Neutered Male	3	1	2
Bodyweight (kg) *	2.4 (1.2–4.7)	2.6 (1.2–4.7)	2.4 (1.8–4.6)
Temperature (°F) *	103 (98–104)	101 (98–104)	103 (100–104)
Effusion location			
Abdominal	12	8	4
Pleural	5	1	4
Pericardial	1	0	1

* Presented as median and range for continuous variables.

**Table 2:** Clinicopathologic data at study enrollment. Variables presented as median (range).

	All Cats	GS-441524 Allocated Cats	Remdesivir Allocated Cats
Effusion			
Cell count (/μL)	2540 (900–26,960)	2530 (200–26,960)	2540 (900–11,660)
Protein (g/dL)	6.5 (4.5–8.4)	6.6 (4.5–8.4)	5.8 (5.0–8.0)
Hematologic			
Hematocrit (%)	27 (15–37)	27 (15–37)	26 (19–35)
Lymphocytes (/μL)	930 (98–2266)	889 (209–1687)	1860 (98–2266)
Neutrophils (/μL)	12,151 (5713–52,882)	15,576 (8414–52,882)	11,508 (5713–16,991)
Bands (/μL)	0 (0–2344)	0 (0–960)	0 (0–2344)
Biochemical			
Albumin (g/dL)	2.2 (1.4–3.0)	2.1 (1.6–3.0)	2.3 (1.4–3.0)
Globulin (g/dL)	5.8 (3.3–7.4)	6.0 (4.1–7.4)	5.1 (3.3–7.2)
Albumin:Globulin	0.4 (0.3–0.9)	0.5 (0.3–0.5	0.4 (0.3–0.9)
Bilirubin (mg/dL)	2.0 (0.1–3.7)	0.7 (0.2–3.7)	0.2 (0.1–3.1)

## Data Availability

Patient data has been anonymized for publication and may be available upon request.
